# Post-mortem forensic identity testing: application of PCR to the identification of fire victim

**DOI:** 10.1590/S1516-31802000000300005

**Published:** 2000-05-02

**Authors:** José Arnaldo Soares-Vieira, Ana Elisa Correia Billerbeck, Edna Sadayo Miazato Iwamura, Laís de Almeida Cardoso, Daniel Romero Muñoz

**Keywords:** Fire victims, DNA typing, PCR, Human identification, Paternity investigation, Carbonizados, Tipagem de DNA, PCR, Identificação humana, Investigação de paternidade

## Abstract

**CONTEXT:**

DNA analysis has been used with success in the identification of carbonized corpses and victims of large accidents. The analysis requires relatives of crash victims to donate blood for analysis. The relatives are generally willing contribute to the identification by giving a blood sample.

**OBJECTIVE:**

To describe the use of the polymerase chain reaction (PCR) for genetic characterization of one victim extensively burned by fire.

**DESIGN:**

Case report.

**CASE REPORT:**

DNA was extracted from blood of the cardiac chamber, and 15 different *loci* (D1S80, ApoB, D17S30, D3S1744, D18S849, D12S1090, FGA, D7S820, D1S533, D9S304, HUMCSF1PO, HUMTPOX, HUMTHO1, amelogenin and HLA-DQA1) were analyzed using the PCR technique. Results from all loci typing of the corpse were then compared to that of his alleged biological parents, revealing a genetic compatibility.

## resumo

**CONTEXTO:**

A análise do DNA tem sido utilizada com sucesso na identificação de carbonizados e de vítimas de grandes acidentes. A análise do DNA necessita de amostras de sangue dos familiares das vítimas como referência. Os familiares normalmente contribuem com a identificação doando uma amostra de sangue.

**OBJETIVO:**

Este trabalho descreve o uso da reação em cadeia de polimerase (PCR) para caracterização genética de uma vítima extensamente queimada pelo fogo.

**TIPO DE ESTUDO:**

Relato de caso

**RELATO DE CASO:**

O DNA foi extraído de sangue de câmara cardíaca e 15 *loci* (D1S80, ApoB, D17S30, D3S1744, D18S849, D12S1090, FGA, D7S820, D1S533, D9S304, HUMCSF1PO, HUMTPOX, HUMTHO1, amelogenina e HLA-DQA1) foram analisados utilizando-se a técnica da PCR. Os resultados de todos os loci analisados a partir de DNA genômico extraído do corpo carbonizado foram comparados com os resultados obtidos a partir de DNA genômico extraído de sangue dos supostos pais biológicos do corpo, revelando uma compatibilidade genética.

## INTRODUCTION

DNA typing techniques are one of the most advanced tools for human identification.^1^ During the last 10 years, a great number of methods for DNA typing have been introduced to forensic science, with considerable success and also with considerable controversy.^2–4^ The success and validation of a criminal investigation are very closely related to the process used for obtaining and preserving biological evidence.^5^

Genetic markers can be characterized in traces of biological fluids, such as blood,^6,7^ sperm,^8^ saliva,^9,10^ nasal secretion,^11^ fragmented skeletal remains and old bones.^12–15^

In severely charred fire victims both autolytic changes as well as deleterious effects of heat will cause degradation of the DNA.^16^

Polymerase chain reaction (PCR) procedures permit reliable replication of thousand of copies of a specific DNA sequence, *in vitro*, and have been described and improved in recent years,^17–19^ allowing the study of small amounts of DNA even when degraded. These procedures are therefore extremely useful in the analysis of forensic samples. Several *loci* are especially suitable for PCR analysis. DNA analysis has been used with success in the identification of carbonized corpses and victims of large accidents.^16,20,21^ The analysis does, however, require relatives of crash victims to donate blood for analysis. It is found that the relatives are generally willing to contribute to the identification by giving a blood sample.^21^

This paper describes the use of PCR for genetic characterization of one victim extensively burned by fire. This identification was possible by analyzing the DNA extracted from blood of the cardiac chamber of this corpse.

## CASE REPORT

### Scenario

During a recent police chase following a kidnapping, a car crashed and burned. An intensely burned corpse was removed from the car and sent for forensic identification. There were no fingerprints or other ways to identify the corpse, but there was a suspicion about its identity. To confirm this hypothesis, the DNA of the alleged parents was compared to the DNA extracted from the burned corpse.

### Procedures

DNA was extracted from blood of the cardiac chamber of the carbonized corpse by the Kunkel method^22^ and from 5 ml of peripheral blood obtained from the alleged biological parents of the corpse by the salting–out procedure.^23^ The D1S80 *locus* was studied using the D1S80 Forensic DNA Amplification Reagent Kit (Perkin Elmer, USA), as recommended by the manufacturer. The amplified fragments were submitted to electrophoresis on a polyacrylamide gel (GeneAmp Detection Gel – Perkin Elmer) and visualized after silver staining. Allele identification was achieved by comparison of the amplified fragments to the allelic ladder included in the kit. The study of the HLA-DQA1 *locus* and the analysis of the alleles were performed using the Amplitype HLA-DQA1 Forensic DNA Amplification and Typing Kit (Perkin Elmer, USA), as recommended by the manufacturer. The amplification and analysis of the D3S1744, D18S849, D12S1090, FGA, D7S820, D1S533 and D9S304 *loci* were assembled using the components of the Multiplex I and Multiplex II Kits (Lifecodes Corp., USA). The CTT Multiplex Kit and Amelogenin Kit (Promega Corp., USA) was used for the amplification and study of the HUMCSF1PO, HUMTPOX, HUMTH01 and amelogenin *loci* . The Multiplex and amelogenin kits were used as recommended by the manufacturer. The D17S30 *locus* was studied using primers, reactions and gel analysis as described by Horn.^24^ Allele identification was achieved by comparison of the amplified fragments to the allelic ladder (Promega Corp., USA). The ApoB *locus* was studied using primers, reactions and gel analysis as described by Boerwinkle.^25^ Allele identification was achieved by comparison of the amplified fragments to the 100 bp and 123 bp ladder (GIBCO-BRL, USA). The amplification products obtained from genomic DNA extracted from the blood of the corpse were compared to those generated from genomic DNA obtained from corpse's alleged biological parents.

The data obtained from the study of the 15 *loci* are presented in Table.

**Table:** Genotypes identified in the three DNA samples

Locus	AF	AM	CC	RESULTS
HLA-DQA1	4, 4	1.1, 4	1.1, 4	No exclusion
D17S30	4, 5	12, 12	5, 12	No exclusion
ApoB	A, B	B, C	B, C	No exclusion
D1S80	20, 31	21, 24	20, 24	No exclusion
HUMCSF1PO	9, 10	10, 11	9, 11	No exclusion
HUMTPOX	8, 9	7, 8	7, 8	No exclusion
Amelogenin	XY	XX	XY	–
HUMTHO1	9, 10	6, 8	8, 9	No exclusion
D3S1744	18, 19	18, 20	18, 19	No exclusion
D18S849	17, 18	9, 9	9, 17	No exclusion
D12S1090	9, 13	11, 13	11, 13	No exclusion
FGA	25, 26	24, 24	24, 26	No exclusion
D7S820	7, 10	9, 12	9, 10	No exclusion
D1S533	13, 14	15, 15	13, 15	No exclusion
D9S304	8, 12	12, 12	12, 12	No exclusion

AF: alleged father of carbonized corpse; AM: alleged mother of carbonized corpse; CC: carbonized corpse.

## DISCUSSION

The identification of remains from fire victims is generally attempted by recognizing personal effects, individualizing marks (e.g. scars, tattoos, signs of known disease) and/or dental records.

However, due the effects of heat and severe laceration of the body, the identification by usual forensic means is not always possible. In such cases DNA may be obtained from crude tissue samples (about 1 gram), collected during autopsy,^16^ from soft tissues within the nerve chamber of unerupted wisdom teeth^20^ or from muscles.^21^ A paternity test was performed and Mendelian inheritance of the alleles for these 15 *loci* was observed.

The present study revealed that biological material (blood) collected from the cardiac chamber of the carbonized corpse, even with highly degraded DNA, could be analyzed by the PCR technique and positively identify the victim.

Professional workers, like detectives, prosecutors, lawyers, judges, doctors etc., who act in different forensic areas related to the solution of crimes and human identification have long had the need for an efficient method capable of positively identifying an individual. Recent advances in molecular biology have significantly enhanced the potential for individualization by providing a capability for typing DNA from various biological materials. DNA analysis has been used successfully in a number of large accidents to associate body parts and for the purposes of identification, by comparing victims’ DNA profiles with those of relatives. In this sense, molecular biology has become an important and fundamental tool for personal investigation.

## References

[1] Jeffreys AJ, Brookfield JFY, Semeonoff R (1985). Positive identification of an immigration test case using human DNA fingerprints. Nature.

[2] Lee HC, Ladd C, Bourke MT, Pagliaro EM, Tirnady F (1994). DNA typing in forensic science. Am J Forensic Med Pathol.

[3] Committee on DNA Technology in Forensic Science, National Research Council (1992). DNA technology in forensic science.

[4] Committee on DNA Forensic Science, National Research Council (1996). The evaluation of forensic DNA evidence.

[5] Lee HC, Ladd C, Scherczinger CA, Bourke MT (1998). Forensic applications of DNA typing. Part 2: Collection and preservation of DNA evidence. Am J Forensic Med Pathol.

[6] Stein C, Kyeck SH, Henssge C (1996). DNA typing of fingerprint reagent treated biological stains. J Forensic Sci.

[7] Soares-Vieira JA As aplicações da biologia molecular na identificação humana em manchas e crostas de sangue. São Paulo, 1998.

[8] Laperche S, Van Huffel V, Rouger P, Salmon C (1991). Restriction fragment study (RFLP) of DNA polymorphism in criminology: quantitative and qualitative analysis beginning with dried blood and semen on various supports. Rev Fr Transfus Hemobiol.

[9] Hochmeister MN, Budowle B, Jung J, Borer UV, Comey CT, Dirnhofer R (1991). PCR-based typing of DNA extracted from cigarette butts. Int J Leg Med.

[10] Sweet D, Lorente M, Valenzuela A, Lorente JA, Alvarez JC (1996). Increasing DNA extraction yield from saliva stains with a modified Chelex method. Forensic Sci Int.

[11] Tahir MA, Caruso JF, Hamby PP, Sovinski SM, Tahir UA (1995). Restriction fragment length polymorphism (RFLP) typing of DNA extracted from nasal secretions. J Forensic Sci.

[12] Holland MM, Fisher DL, Mitchell LG (1993). Mitochondrial DNA sequence analysis of human skeletal remains: identification of remains from the Vietnam War. J Forensic Sci.

[13] Gill P, Ivanov PL, Kimpton C (1994). Identification of the remains of the Romanov family by DNA analysis. Nature Genet.

[14] Boles TC, Snow CC, Stover E (1995). Forensic DNA testing on skeletal remains from mass graves: a pilot project in Guatemala. J Forensic Sci.

[15] Corach D, Sala A, Penacino G (1997). Additional approaches to DNA typing of skeletal remains: the search for "missing" persons killed during the last dictatorship in Argentina. Electrophoresis.

[16] Sajantila A, Strom M, Budowle B, Karhunen PJ, Peltonen L (1991). The polymerase chain reaction and post-mortem forensic identity testing: application of amplified D1S80 and HLA-DQa loci to the identification of fire victims. Forensic Sci Int.

[17] Erlich HA, Gelfand DH, Saiki RK (1988). Specific DNA amplification. Nature.

[18] Marx JL (1988). Multiplying genes by leaps and bounds. Science.

[19] Saiki RK, Gelfand DH, Stoffel S (1988). Primer-directed enzymatic amplification of DNA with a thermostable DNA polymerase. Science.

[20] Sweet DJ, Sweet HW (1995). DNA analysis of dental pulp to link incinerated remains of homicide victim to crime scene. J Forensic Sci.

[21] Olaisen B, Stenersen M, Mevag B (1997). Identification by DNA analysis of the victims of the August 1996 Spitsbergen civil aircraft disaster. Nature Genetics.

[22] Kunkel LM, Smith KD, Boyer SH (1977). Analysis of human Ychromosome –specific reiterated DNA in chromosome variants. Proc Natl Acad Sci.

[23] Miller SA, Dykes DD, Polesky HF (1988). A simple salting-out procedure for extracting DNA from human nucleated cells. Nucleic Acids Res.

[24] Horn GT, Richards B, Klinger KW (1989). Amplification of a highly polymorphic VNTR segment by the polymerase chain reaction. Nucleic Acids Res.

[25] Boerwinkle E, Xiong W, Fourest E, Chan L (1989). Rapid typing of tandemly repeated hypervariable loci by the polymerase chain reaction: Application to the apolipoprotein B 3’ hypervariable region. Proc Natl Acad Sci USA.

